# Synthesis of molecularly imprinted polymers using a functionalized initiator for chiral‐selective recognition of propranolol

**DOI:** 10.1002/chir.23167

**Published:** 2020-01-13

**Authors:** Weifeng Liu, Clovia Holdsworth, Lei Ye

**Affiliations:** ^1^ Division of Pure and Applied Biochemistry, Department of Chemistry Lund University Lund Sweden; ^2^ Key Laboratory of Interface Science and Engineering in Advanced Materials, Ministry of Education Taiyuan University of Technology Taiyuan China; ^3^ Discipline of Chemistry, School of Environmental and Life Sciences University of Newcastle Callaghan New South Wales Australia

**Keywords:** chiral selectivity, functional initiator, molecular imprinting, molecular recognition, propranolol

## Abstract

We present a new concept of synthesis for preparation of molecularly imprinted polymers using a functionalized initiator to replace the traditional functional monomer. Using propranolol as a model template, a carboxyl‐functionalized radical initiator was demonstrated to lead to high‐selectivity polymer particles prepared in a standard precipitation polymerization system. When a single enantiomer of propranolol was used as template, the imprinted polymer particles exhibited clear chiral selectivity in an equilibrium binding experiment. Unlike the previous molecular imprinting systems where the active free radicals can be distant from the template‐functional monomer complex, the method reported in this work makes sure that the actual radical polymerization takes place in the vicinity of the template‐associated functional groups. The success of using functional initiator to synthesize molecularly imprinted polymers brings in new possibilities to improve the functional performance of molecularly imprinted synthetic receptors.

## Graphical abstract

Formation of specific binding sites in molecularly imprinted polymers can be achieved by using a functional initiator in simplified reaction system.

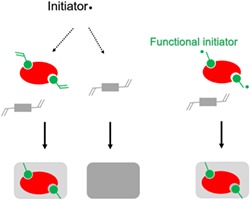

## 1 INTRODUCTION

Owing to the specific molecular recognition capability, molecularly imprinted polymers (MIPs) have attracted enormous interest in applied science and technology areas. Molecular imprinting has been widely used to prepare robust polymer materials with predefined molecular selectivity for applications such as affinity‐based separations, solid phase extraction in analytical sample preparation, controlled delivery of therapeutic drugs, and chemical sensors.^1^, ^2^, ^3^, ^4^ Molecular imprinting technique involves the preparation of a synthetic polymer through the cross‐linking of functional monomers in the presence of a molecular template. In the first step, a template‐functional monomer complex is formed through covalent or non‐covalent interactions. Subsequently, the functional monomer is copolymerized with a cross‐linking monomer to form a solid polymer matrix. After removal of the template molecule, a molecular binding site is left, with its shape, size, and interacting groups defined by the original template. After a successful molecular imprinting, the molecular binding sites in the MIP will be able to recognize and bind the original template with a very high selectivity.

Different polymerization methods have been developed to prepare MIPs, such as bulk polymerization, suspension polymerization, precipitation polymerization, and polymerization on a solid surface.^5^, ^6^, ^7^, ^8^ Of all the methods developed to synthesize MIPs, one critical issue is to select the correct functional monomer, because the strength of the template‐functional monomer interactions often determines the quality of the final imprinted sites. The template‐functional monomer complex can be stabilized by various interactions including covalent bonding, hydrogen bond and metal ion coordination.^9^, ^10^, ^11^ In the non‐covalent imprinting approach, methacrylic acid (MAA) is frequently used as a functional monomer because its carboxyl group can act as both hydrogen bond donor and acceptor to bind different template molecules. As examples, MAA has been used successfully as functional monomer to synthesize propranolol‐imprinted polymer beads by suspension polymerization,^12^ Pickering emulsion polymerization,^13^, ^14^ and precipitation polymerization.^15^ The simplicity and straightforward synthesis by precipitation polymerization is of particular interest, as it provides an ideal system to gain mechanistic insight into molecular imprinting process using easily accessible analytical tools, eg, solution NMR spectroscopy and dynamic light scattering.^16^

**Figure 1:**
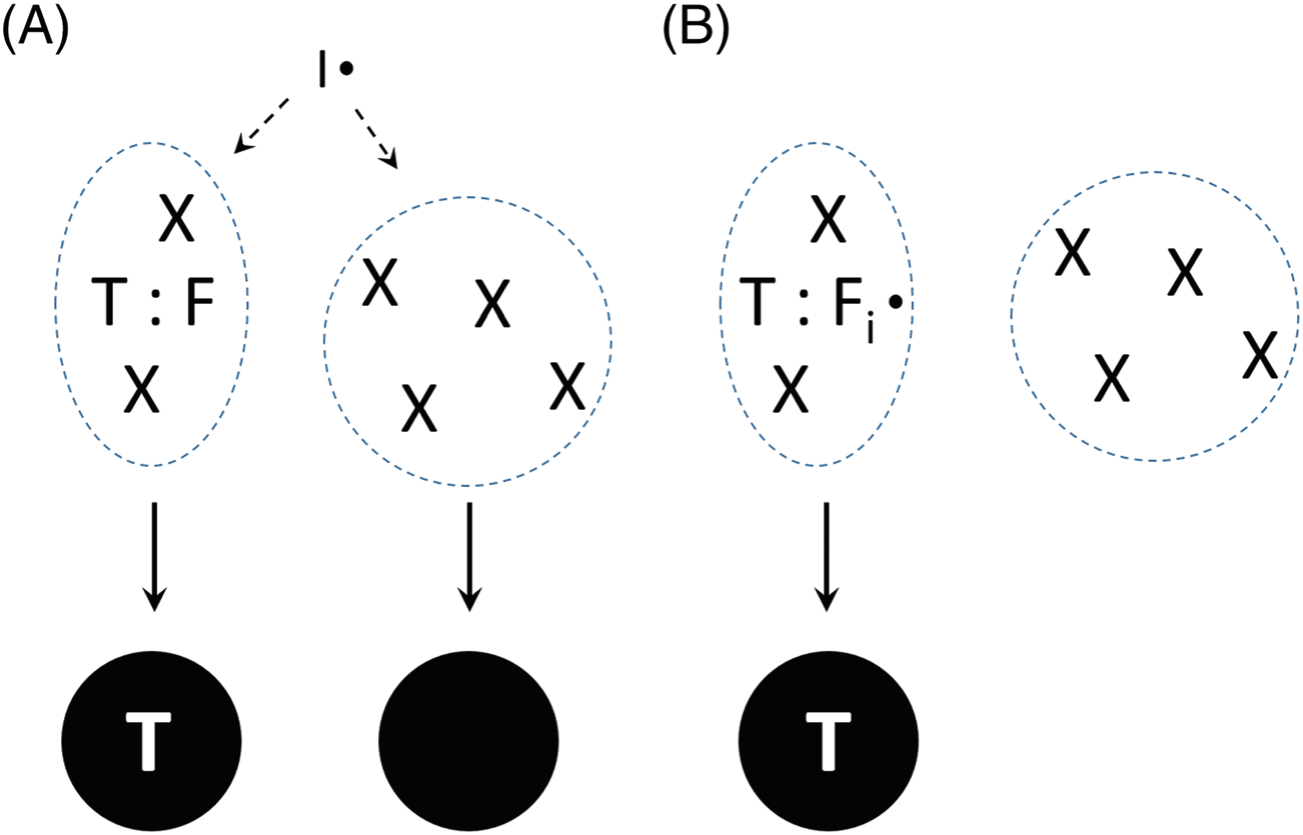
Formation of specific MIP particles in precipitation polymerization system using (A) traditional radical initiator and (B) functionalized radical initiator. T: template; F: functional monomer; X: cross‐linking monomer; I: initiator; F_i_: functionalized initiator. Non‐specific particle is represented by the solid circle. For simplicity, only template‐bound functional monomer and functionalized initiator are presented

A significant part of research in molecular imprinting has been focused on increasing the strength of intermolecular interactions between template and functional monomers.^9^, ^17^, ^18^ In contrast, there is barely any report discussing the impact of radical initiator on molecular imprinting effect.^19^ Taking precipitation polymerization into account, under a standard molecular imprinting condition, one can expect that a significant portion of cross‐linking reaction can lead to non‐specific particles, because the active radicals generated from the initiator have no affinity for the template (Figure 1A). Only when an active radical reacts with the template‐bound functional monomer, an imprinted molecular binding site will start to form (Figure 1A). Based on this consideration, we propose that a functionalized initiator may be used to prepare MIPs without requiring commonly used functional monomers. Besides simplifying the reaction components, the functionalized initiator may help to reduce the amount of non‐specific polymer particles, because the cross‐linking reaction will take place in the vicinity of the bound molecular template, leading to formation of template‐imprinted sites (Figure 1B).

**Figure 2:**
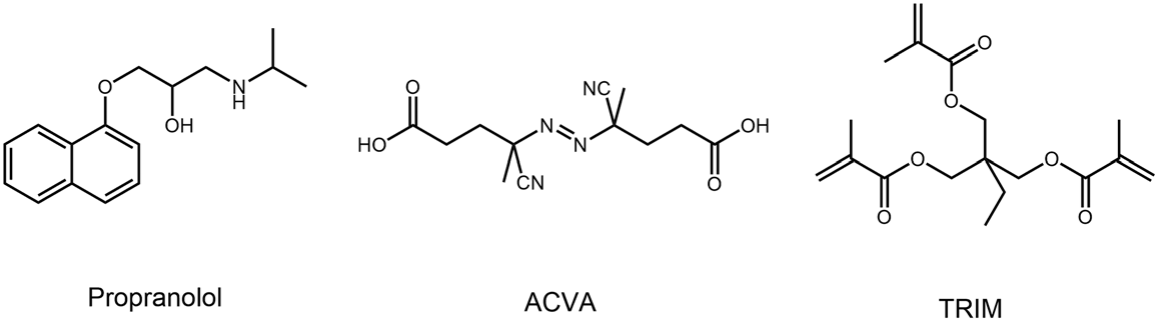
Chemical structures of propranolol, functional initiator (ACVA), and cross‐linking monomer (TRIM) used in this work

In this work, we use a well‐investigated molecular template propranolol to demonstrate the feasibility of non‐covalent molecular imprinting using functionalized initiator (Figure 2). Propranolol was chosen as the model because it is a chiral therapeutic drug. Propranolol enantiomer is commercially available and can be used as a probe to investigate chiral selectivity of the imprinted polymers. As functionalized initiator, we decided to use 4,4′‐azobis(4‐cyanovaleric) acid (ACVA)^20^ because its carboxyl group can form hydrogen bond interactions with propranolol. Trimethylolpropane trimethacrylate (TRIM) was used as the cross‐linking monomer. The precipitation polymerization was carried out in acetonitrile. The polymer particles were purified following standard workup procedures before the molecular recognition characteristics were investigated.

## 2 MATERIALS AND METHODS

### 2.1 Materials

(*R*,*S*)‐Propranolol hydrochloride (99%), (*S*)‐propranolol hydrochloride (99%), and (*R*)‐propranolol hydrochloride (99%) supplied by Fluka (Dorset, UK) were converted into the free base form before use. Trimethylolpropane trimethacrylate (TRIM, technical grade), pindolol (98%), metoprolol (97%), atenolol (98%), and timolol (98%) were purchased from Sigma‐Aldrich (Dorset, UK). Methanol (≥ 99.9%), acetic acid (glacial, 100%), acetone (98%), acetonitrile (99.7%), and 4,4 ′‐azobis(4‐cyanovaleric) acid (98%, ACVA) were purchased from Merck (Darmstadt, Germany). ACVA was recrystallized from methanol before use. Other chemicals were analytical grade and were used as received.

### 2.2 Preparation of MIP particles

MIP particles were synthesized using one‐step precipitation polymerization, as shown in Figure 2. Briefly, the template molecule, (*R*,*S*)‐propranolol (34.25 mg, 0.13 mmol) was dissolved in 10‐mL acetonitrile in a one‐neck round bottomed flask. After adding 74.3 mg (0.27 mmol) of ACVA and 161 μL (0.51 mmol) of TRIM, the solution was purged with a gentle flow of nitrogen gas for 5 minutes and then sealed. The one‐neck round bottomed flask was transferred into a Stovall HO‐10 Hybridization Oven. Then the polymerization was carried out at 60°C for 24 hours while the flask was rotated at a speed of 20 rpm. Finally, the polymer particles were collected by centrifugation, washed with methanol/acetic acid (9:1, v/v) repeatedly to remove the template. The polymer particles were finally washed with acetone and dried in a vacuum desiccator overnight. Non‐imprinted polymer (NIP) was synthesized following the same procedure without adding the template. An enantiomer‐imprinted MIP (sMIP) was synthesized using (*S*)‐propranolol as the template under the same condition.

### 2.3 Characterization

Field emission scanning electron microscopy (FESEM; JSM‐6700F, operated at 10 kV; Japan) and Fourier transformation infrared spectrometry (FT‐IR; Nicolet iS5; USA) were used to characterize the morphologies and structures of the samples. Fluorescence spectrometer (Quanta Master C‐60/2000, excitation wavelength at 292 nm; USA) was used to determine the binding properties of the MIP and NIP particles.

### 2.4 Binding experiments

#### 2.4.1 Kinetic binding experiments

MIP or NIP particles (2.0 mg) were added to 2.0 mL of (*R*,*S*)‐propranolol solution with an initial concentration (*C*_0_) of 10.0μM. The mixtures were stirred on a rocking table at room temperature. At different times, the samples were centrifuged to sediment the polymer particles, and the concentration (*C*_*t*_) of the free (*R*,*S*)‐propranolol in the supernatant was determined using fluorescence spectrometer.

The amount of bound propranolol (*Q*, mg g^−1^) was calculated according to Equation (1):

(1) $$Q=\frac{V\left(C_{0}-C_{t}\right)}{m}\times 10^{3}M_{r},$$

where *V* (mL) and *m* (mg) are the volume of the (*R*,*S*)‐propranolol solution and the mass of the MIP or NIP particles and *M*_*r*_ is the molecular weight of (*R*,*S*)‐propranolol.

#### 2.4.2 Binding isotherm

Six aliquots of MIP or NIP particles (2.0 mg) were introduced into separate microcentrifuge tubes. Then 2.0 mL of (*R*,*S*)‐propranolol solution with concentration (*C*_0_) of 5.0μM, 10.0μM, 20.0μM, 40.0μM, 60.0μM, 80.0μM, and 100.0μM was added into each centrifuge tube. The samples were stirred on a rocking table at room temperature. After stirring for 90 minutes, the equilibrium concentration of the unbound (*R*,*S*)‐propranolol (*C*_*e*_, μM) was determined by fluorescence spectrometer. The equilibrium binding (*Q*_*e*_, mg g^−1^) was calculated according to Equation (2), 21, 22:

(2) $$Q_{e}=\frac{V\left(C_{0}-C_{e}\right)}{m}\times 10^{3}M_{r}$$

#### 2.4.3 Regeneration and reusability of MIP particles

MIP or NIP particles (2.0 mg) were added to a solution of (*R*,*S*)‐propranolol in 2.0 mL of acetonitrile with a concentration of 10.0μM. After incubation for 90 minutes at room temperature, the amount of free (*R*,*S*)‐propranolol was quantified by fluorescence spectrometer. The MIP or NIP particles were subsequently washed with methanol/acetic acid (9:1, v/v) to remove the bound (*R*,*S*)‐propranolol. The polymer particles were finally washed with acetone and dried in a vacuum desiccator. The adsorption and desorption cycle was repeated using the regenerated MIP or NIP particles.

### 2.5 Chiral selectivity of enantiomer‐imprinted polymer

(*S*)‐Propranolol‐imprinted polymer particles (sMIP) or NIP particles (2.0 mg) were added into an enantiomer solution of (*S*)‐ or (*R*)‐propranolol in 2.0 mL of acetonitrile with a concentration of 10.0μM. After incubation for 90 minutes at room temperature, the amount of (*S*)‐ or (*R*)‐propranolol bound to sMIP and NIP particles was quantified by fluorescence intensity measurement.

## 3 RESULTS AND DISCUSSION

### 3.1 Characterization of MIP particles

**Figure 3:**
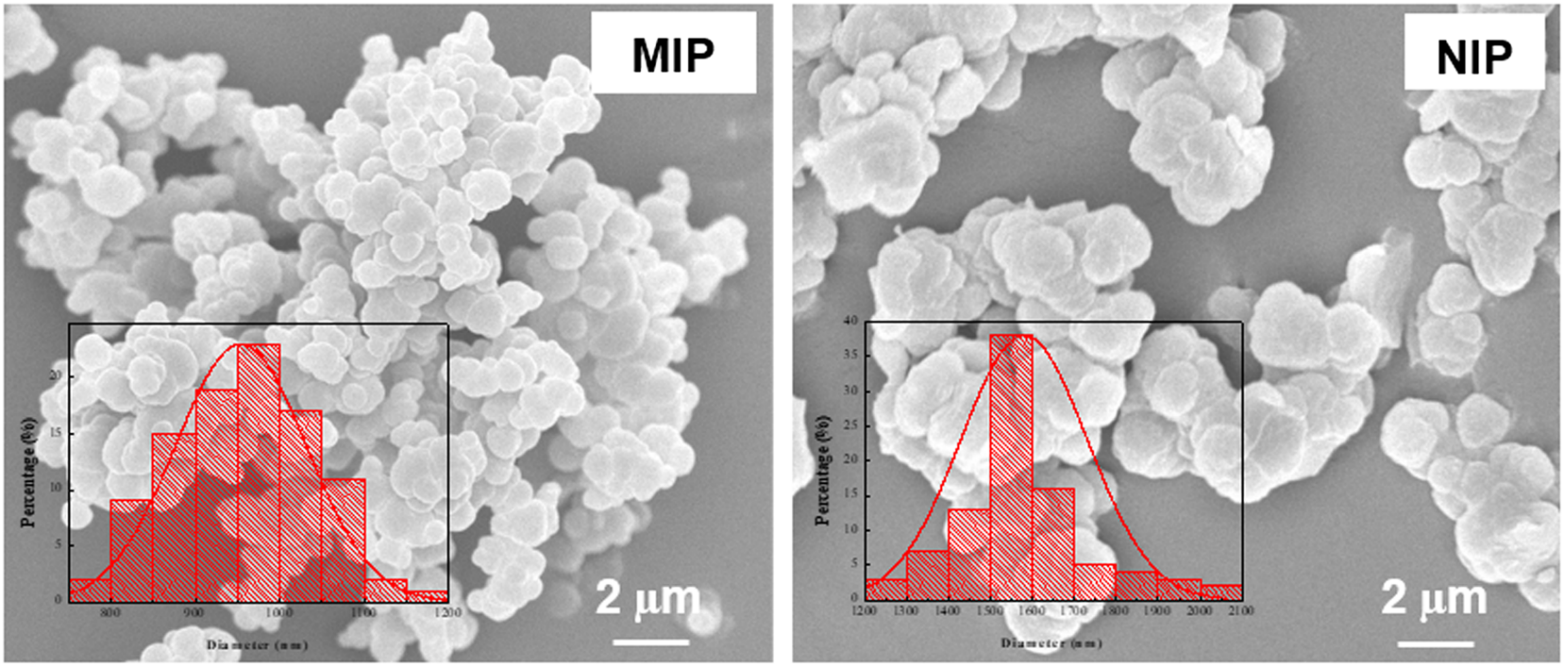
SEM images of MIP and NIP particles prepared using ACVA as functionalized initiator

Using ACVA as a functionalized initiator and TRIM as the cross‐linking monomer, the molecular imprinting reaction carried out in acetonitrile resulted in microspheres with an average diameter of 0.95 μm, as shown in Figure 3. Under the same polymerization condition, the NIP particles are larger, with an average diameter of 1.6 μm as measured from the SEM image (Figure 3). The difference of particle size between the MIP and NIP particles is due to the presence of the template molecule in the imprinting system. This phenomenon has been observed in our previous studies where propranolol was used as the molecular template, and MAA was used as functional monomer to prepare MIP microspheres by precipitation polymerization.^15^ The presence of propranolol in the reaction mixture affected the particle nucleation and growth, thereby caused the MIP particles to become smaller.

**Figure 4:**
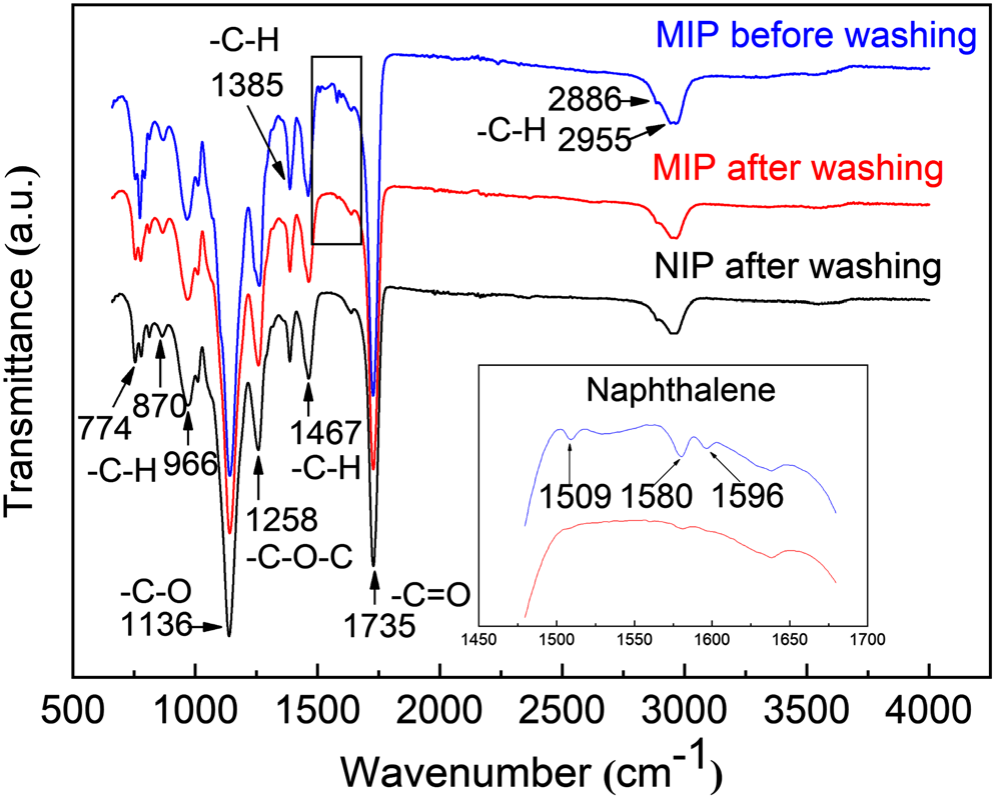
FT‐IR spectra of NIP particles and MIP particles before and after template removal

FT‐IR analysis was used to investigate the functional groups in the MIP and NIP particles (Figure 4). For all the polymer samples, obvious absorption bands at 2955, 2886, 1735, 1467, 1385, 1258, 1136, 966, 870, and 774 cm^−1^ are observed and can be assigned to the –C‐H, –C=O, –O‐H and –C‐O structures, which are from the functional initiator ACVA and the cross‐linking monomer TRIM. Compared with the FT‐IR spectra of ACVA and TRIM shown in Figure S1, the –C=C band at 1636 cm^−1^ from the polymer particles has weakened substantially, suggesting that the cross‐linking polymerization has taken place during the imprinting reaction. The change of IR spectrum of the MIP particles before and after template removal, in particular for the naphthalene bands at 1596, 1580, and 1509 cm^−1^, indicates that the template has been removed after the washing steps.^23^

### 3.2 Binding characteristics of MIP particles

#### 3.2.1 Binding kinetics

**Figure 5:**
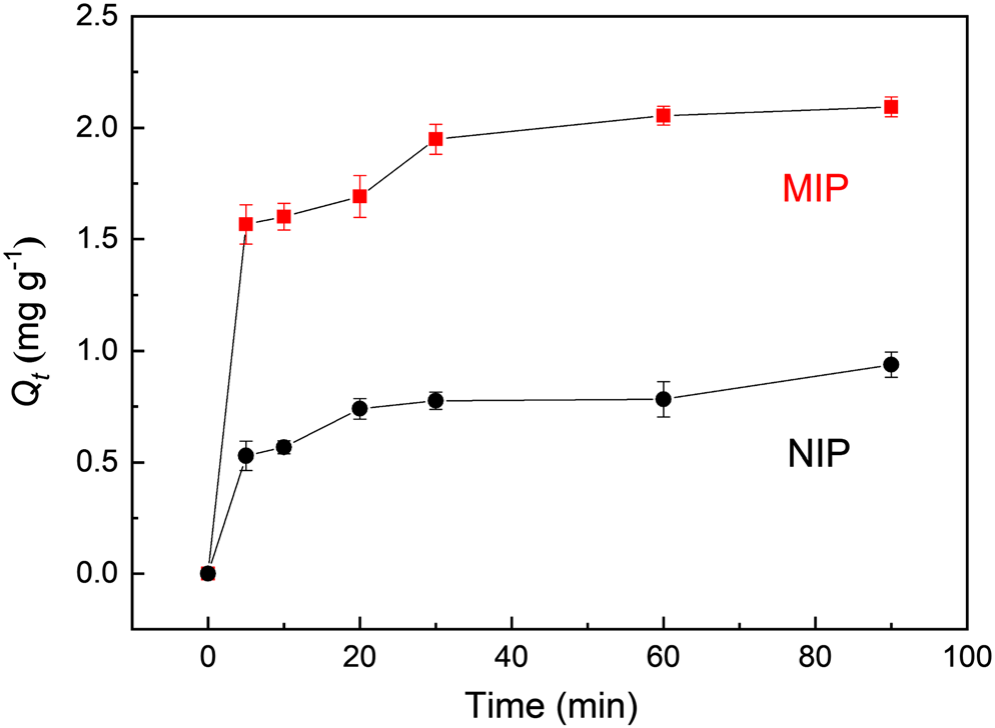
Kinetic binding curves of MIP and NIP for adsorption of propranolol in acetonitrile

The kinetics of template binding is shown in Figure 5. In the initial stage, the amount of propranolol bound increases quickly. After 30 minutes, the uptake of propranolol by MIP and NIP increases more slowly. At the beginning, because of the abundance of unoccupied imprinted cavities, the template molecules can be easily taken up by the cavities present on the polymer surface, leading to a rapid adsorption of propranolol.^24^ After 90 minutes, the propranolol binding to the MIP particles reached the equilibrium value of 2.10 mg g^−1^. The NIP particles exhibit similar kinetic profile but have a lower equilibrium binding (0.94 mg g^−1^) due to the absence of imprinted cavities.^25^ For the NIP particles, owing to the randomly distributed functional groups, some propranolol binding was observed.

To study the kinetics of propranolol binding with MIP and NIP, pseudo‐first‐order and pseudo‐second‐order kinetic models (Equations 3 and 4) were used to fit the experimental data:^26^, ^27^

(3) $$\mathrm{lg}\left(Q_{e}-Q_{t}\right)=\mathrm{lg}Q_{e}-\frac{k_{1}}{2.303}\;t,$$

(4) $$\frac{t}{Q_{t}}=\frac{1}{k_{2}Q_{e}^{2}}+\frac{t}{Q_{e}},$$

where *Q*_*e*_ (mg g^−1^) and *Q*_*t*_ (mg g^−1^) are the amount of adsorption at equilibrium and the amount of adsorption at time *t* (min), respectively. *k*_1_ (min^−1^) and *k*_2_ (g mg^−1^ min^−1^) are the pseudo‐first‐order and pseudo‐second‐order rate constants of adsorption, respectively.

The linear fitting results are shown in Figure S2, and the calculated results are presented in Table S1. The values of correlation coefficient (*R* ^2^) of pseudo‐second‐order model for MIP and NIP are higher than those for the pseudo‐first‐order model and are closer to 1. In addition, the calculated *Q*_*e, cal*_ values from pseudo‐second‐order model are close to the experimental data (*Q*_*e, exp*_). The data illustrate that pseudo‐second‐order mechanism is more suitable to describe the adsorption kinetics than pseudo‐first‐order model, and the potential rate‐limiting step in (*R*,*S*)‐propranolol binding is the chemical adsorption involving specific molecular interactions with the imprinted cavities.^28^, ^29^, ^30^, ^31^

#### 3.2.2 Adsorption isotherm

**Figure 6:**
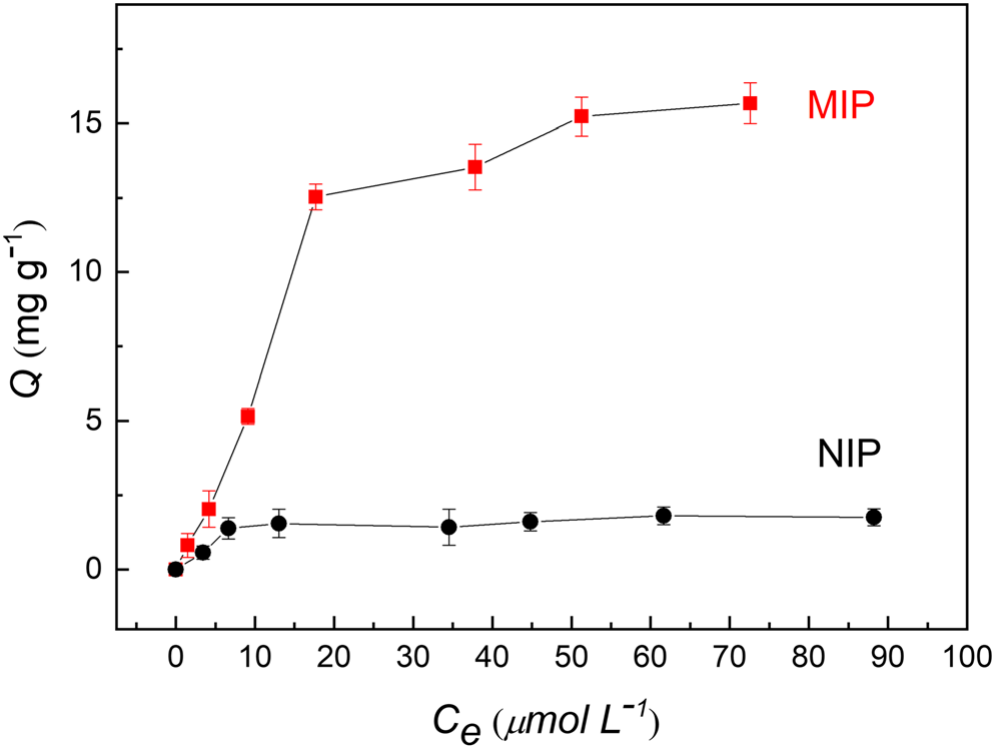
Adsorption isotherm of (*R*,*S*)‐propranolol on MIP and NIP particles measured in acetonitrile

The adsorption isotherm represents the relationship between the equilibrium concentration and the amount of (*R*,*S*)‐propranolol bound to the MIP and NIP particles. As can be seen in Figure 6, with increasing (*R*,*S*)‐propranolol concentration, the equilibrium adsorption on the MIP particles increases rapidly. The amount of (*R*,*S*)‐propranolol bound on the MIP particles is significantly higher than on the NIP particles, suggesting that the imprinted sites in the MIP particles have higher affinity for the template molecule.

Langmuir and Freundlich isothermal models were used to fit the data of equilibrium binding of (*R*,*S*)‐propranolol on MIP and NIP particles. The Langmuir and Freundlich isothermal equations are as follows^26^, ^27^:

(5) $$\frac{C_{e}}{Q_{e}}=\frac{C_{e}}{Q_{m}}+\frac{1}{K_{L}Q_{m}},$$

(6) $$lgQ_{e}=\frac{1}{n}{\mathrm{lgC}}_{e}+{\mathrm{lgK}}_{F},$$

where *C*_*e*_ (μM) and *Q*_*e*_ (mg g^−1^) are the equilibrium concentration and the amount of bound (*R*,*S*)‐propranolol, respectively, *Q*_*m*_ (mg g^−1^) is the maximum adsorption capacity, and *K*_*d*_ is the Langmuir constant. *K*_*F*_ and *n* are the Freundlich adsorption equilibrium constants.

The fitting results are shown in Figure S3 and Table S2. For MIP, the experimental data fit better with the Freundlich isotherm than with the Langmuir model, as indicated by the correlation values (*R* ^2^). The results suggest that the interaction between (*R*,*S*)‐propranolol and the imprinted sites is not homogeneous. For the NIP particles however, the correlation value of the Langmuir fitting (*R* ^2^ = 0.9890) is higher than that of the Freundlich fitting (*R* ^2^ = 0.6347), suggesting that the adsorption of (*R*,*S*)‐propranolol on the NIP takes place mainly on particle surface.

#### 3.2.3 Regeneration

**Figure 7:**
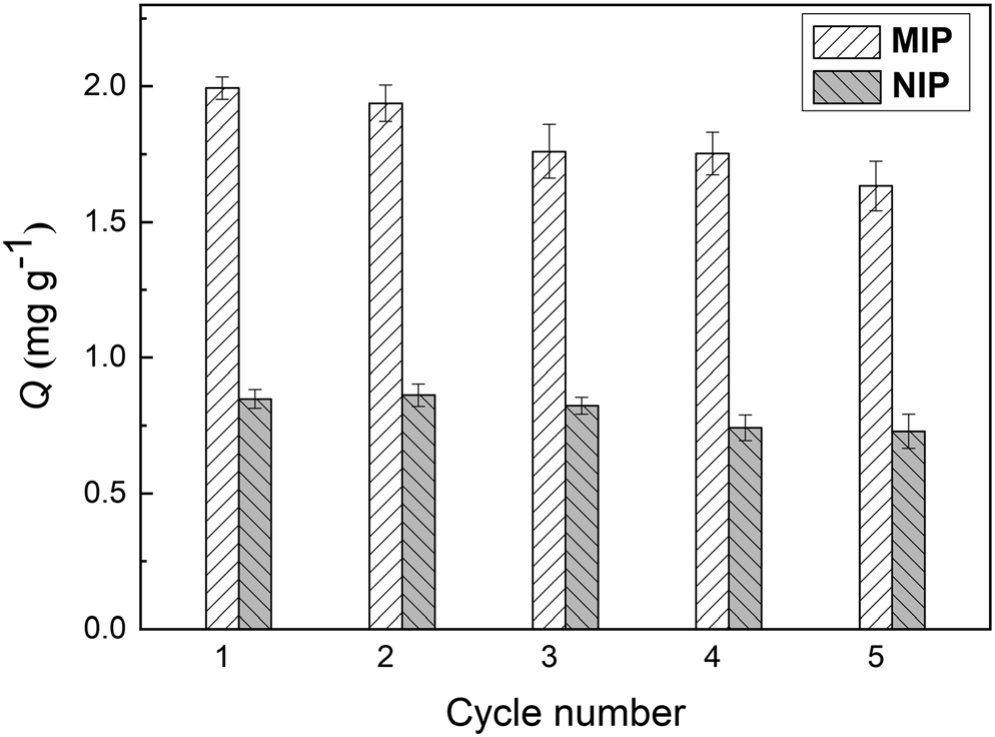
Regeneration of MIP and NIP particles for adsorption (*R*,*S*)‐propranolol in acetonitrile

To investigate the stability and regeneration ability of MIP, adsorption‐desorption cycles were repeated five times using the same polymer particles. As observed in Figure 7, the uptake of (*R*,*S*)‐propranolol only declined slightly with the recycled particles. After five times of use, the (*R*,*S*)‐propranolol binding to the MIP particles remained at a high level (about 82% of the initial binding). Therefore, the imprinted polymer particles are possible to be regenerated and can be reused.

#### 3.2.4 Chiral‐selective molecular recognition

**Figure 8:**
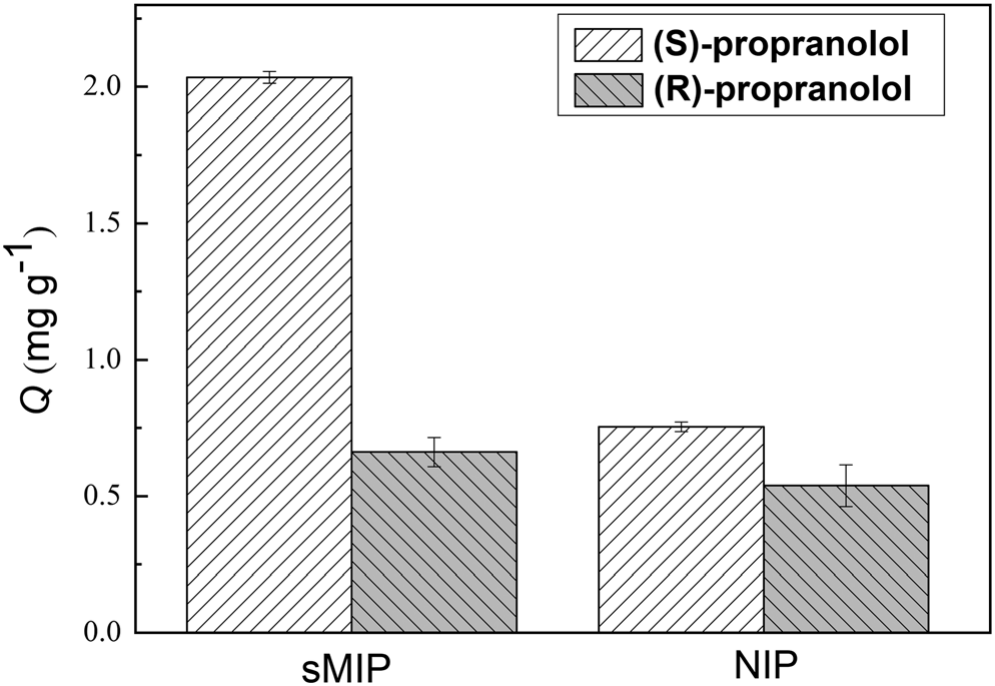
Adsorption capacities of sMIP and NIP for adsorption of (*S*)‐ and (*R*)‐propranolol in acetonitrile

For many chiral pharmacological products, the therapeutic effects of enantiomers can be dramatically different. It is therefore useful to prepare MIPs with chiral‐selective molecular recognition property. To verify that the functional initiator ACVA can be used to produce chiral‐selective MIPs, a new imprinted polymer (sMIP) was synthesized using (*S*)‐propranolol as the template. The structure characterization data (Figure S4) and molecular binding results (Figure S5) suggest that the chiral imprinted polymer was obtained successfully. The adsorption of (*R*)‐ and (*S*)‐propranolol on the sMIP and the NIP particles are shown in Figure 8. It is clear that the adsorption of (*S*)‐propranolol on sMIP (2.03 mg g^−1^) is much higher than the adsorption of (*R*)‐propranolol (0.66 mg g^−1^). The difference of adsorption between the two enantiomers can be attributed to the molecule matching of (*S*)‐propranolol to the imprinted cavities.^32^, ^33^ The imprinted cavities formed by the template (*S*)‐propranolol are not able to accommodate (*R*)‐propranolol due to the spatial distribution of its functional groups. Formation of the chiral‐selective binding sites during the imprinting process is most likely controlled by the hydrogen bond interactions between the carboxyl group in ACVA and the ether, hydroxyl, and amine groups in (*S*)‐propranolol. Owing to the presence of some carboxyl groups on the polymer surface, some (*R*)‐propranolol were found to adsorb on the sMIP and NIP particles. Based on the unambiguous chiral selectivity exhibited by the sMIP, it is obvious that the use of functionalized initiator for noncovalent molecular imprinting can lead to high selectivity MIP materials.

## 4 CONCLUSIONS

In this work, we have developed a new method to synthesize molecularly imprinted polymers using ACVA as both functional monomer and radical initiator. The obtained MIP showed superior selective recognition ability and recyclability for the adsorption of (*R*,*S*)‐propranolol. The adsorption of (*R*,*S*)‐propranolol on the MIP reached equilibrium within 90 minutes, and the adsorption capacity of the MIP was significantly higher than that of NIP. In addition, the single enantiomer‐imprinted sMIP exhibited unambiguous chiral selectivity for (*S*)‐propranolol. This type of chiral‐selective MIPs may be used to achieve chiral separation of enantiomers commonly encountered in production of pharmaceuticals and other fine chemical products.

## Supporting information

FIGURE S1 FT‐IR spectra of ACVA, TRIM and the MIP.
FIGURE S2 Pseudo‐first‐order (a) and pseudo‐second‐order binding kinetics (b) of (R,S)‐propranolol measured on MIP and NIP particles.
Figure S3 Langmuir (a) and Freundlich (b) adsorption isotherms of (R,S)‐propranolol on MIP and NIP particles.
FIGURE S4 SEM image of sMIP and FT‐IR spectra of NIP and sMIP before and after template removal.
FIGURE S5 Adsorption kinetics and binding isotherm of (S)‐propranolol measured on sMIP and NIP in acetonitrile.

TABLE S1 Kinetic parameters of pseudo‐first‐order and pseudo‐second‐order models.
TABLE S2 Parameters of the Langmuir and Freundlich fittings
