# Supplementary figures and images for: Synthesis of molecularly imprinted polymers using a functionalized initiator for chiral‐selective recognition of propranolol
Source: Chirality. 2020 Jan 13;32(3):370–7. doi: 10.1002/chir.23167 (PMC7027874; doi:10.1002/chir.23167)

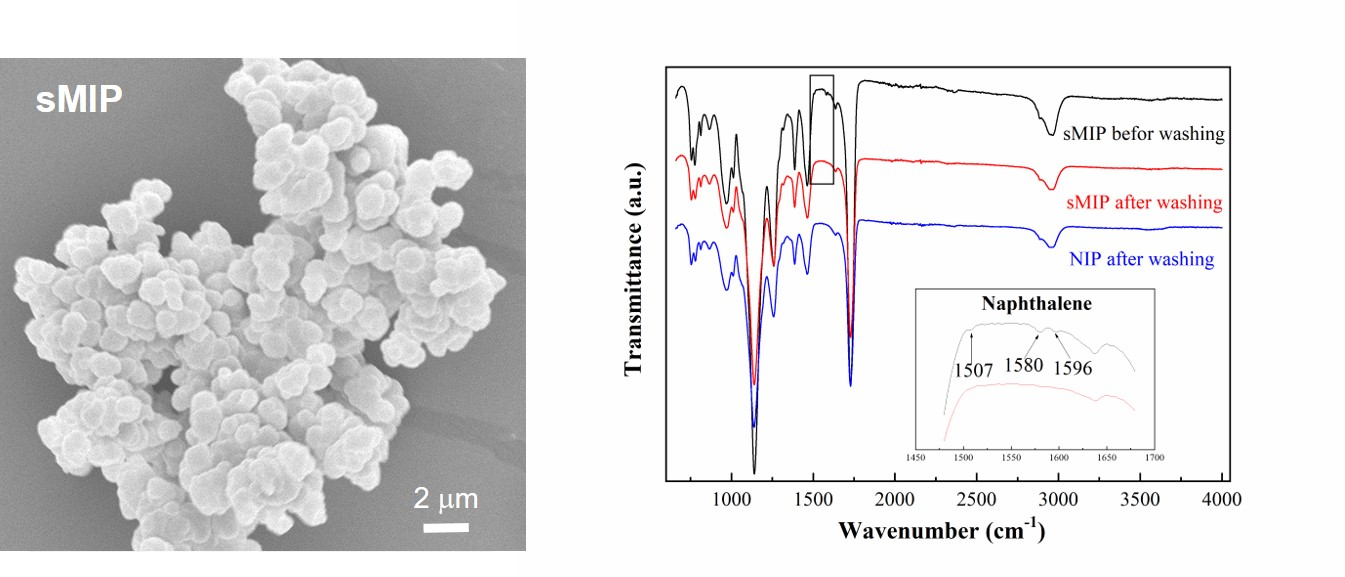

Supplement: Supplementary file 1 — FIGURE S1 FT‐IR spectra of ACVA, TRIM and the MIP. FIGURE S2 Pseudo‐first‐order (a) and pseudo‐second‐order binding kinetics (b) of (R,S)‐propranolol measured on MIP and NIP particles. Figure S3 Langmuir (a) and Freundlich (b) adsorption isotherms of (R,S)‐propranolol on MIP and NIP particles. FIGURE S4 SEM image of sMIP and FT‐IR spectra of NIP and sMIP before and after template removal. FIGURE S5 Adsorption kinetics and binding isotherm of (S)‐propranolol measured on sMIP and NIP in acetonitrile. [file CHIR-32-370-s001.zip › chir23167-sup-0008-Figure_S4.tif]
