# Supplementary material for: Synthesis of molecularly imprinted polymers using a functionalized initiator for chiral‐selective recognition of propranolol
Source: Chirality. 2020 Jan 13;32(3):370–7. doi: 10.1002/chir.23167 (PMC7027874; doi:10.1002/chir.23167)
Supplement: Supplementary file 2 — TABLE S1 Kinetic parameters of pseudo‐first‐order and pseudo‐second‐order models. TABLE S2 Parameters of the Langmuir and Freundlich fittings [file CHIR-32-370-s002.docx]

TABLE S1 Kinetic parameters of pseudo-first-order and pseudo-second-order models

|  | *Q_e, exp_*  (mg g^-1^) | Pseudo-first-order model | | | |  | Pseudo-second-order model | | | |  |
| --- | --- | --- | --- | --- | --- | --- | --- | --- | --- | --- | --- |
|  |  | *Q_e, cal_*  (mg g^-1^) | *k*_1_  (min^-1^) | *R^2^* |  |  | *Q_e, cal_*  (mg g^-1^) | *k*_2_  (g mg^-1^ min^-1^) | *R^2^* |  |  |
| MIP | 2.10 | 1.54 | 0.08475 | 0.9132 |  |  | 2.17 | 0.1297 | 0.9987 | | |
| NIP | 0.94 | 1.29 | 0.08291 | 0.7445 |  |  | 0.96 | 0.1574 | 0.9859 | | |

TABLE S2 Parameters of the Langmuir and Freundlich fittings

|  | Langmuir | | |  | Freundlich | | |
| --- | --- | --- | --- | --- | --- | --- | --- |
|  | *Q_max_* (mg g^-1^) | *K_L_* (L mg^-1^) | *R^2^* |  | *n* | *K_F_* (L g^-1^) | *R^2^* |
| MIP | 25.51 | 0.02674 | 0.8712 |  | 1.2547 | 0.7286 | 0.9346 |
| NIP | 1.860 | 0.1842 | 0.9890 |  | 3.8197 | 0.6067 | 0.6347 |
